# Effects of STN-DBS on cognition and mood in young-onset Parkinson’s disease: a two-year follow-up

**DOI:** 10.3389/fnagi.2023.1177889

**Published:** 2024-01-16

**Authors:** Jun Hong, Huimin Xie, Yuhua Chen, Di Liu, Tianyu Wang, Kun Xiong, Zhiqi Mao

**Affiliations:** ^1^Department of Anatomy and Neurobiology, School of Basic Medical Science, Central South University, Changsha, China; ^2^Department of Neurosurgery, The First Medical Centre, Chinese PLA General Hospital, Beijing, China; ^3^Hebei Key Laboratory of Nerve Injury and Repair, Chengde Medical University, Chengde, China; ^4^Key Laboratory of Emergency and Trauma, Ministry of Education, College of Emergency and Trauma, Hainan Medical University, Haikou, China; ^5^Hunan Key Laboratory of Ophthalmology, Central South University, Changsha, China

**Keywords:** Parkinson’s disease, deep brain stimulation, cognition, verbal fluency, mood

## Abstract

**Background:**

The effects of subthalamic nucleus deep brain stimulation (STN-DBS) on the cognition and mood of patients with PD are still not uniformly concluded, and young-onset Parkinson’s disease (YOPD) is even less explored.

**Objective:**

To observe the effectiveness of STN-DBS on the cognition and mood of YOPD patients.

**Methods:**

A total of 27 subjects, with a mean age at onset of 39.48 ± 6.24 and age at surgery for STN-DBS of 48.44 ± 4.85, were followed up preoperatively and for 2 years postoperatively. Using the Unified Parkinson disease rating scale (UPDRS), H&Y(Hoehn and Yahr stage), 39-Item Parkinson’s Disease Questionnaire (PDQ-39), Mini-mental state examination (MMSE), Montreal Cognitive Assessment (MoCA), Hamilton depression scale (HAMD), Hamilton anxiety scale (HAMA) to assess motor, cognition, and mood.

**Results:**

At the 2-year follow-up after STN-DBS, YOPD patients showed significant improvements in motor and quality of life (UPDRS III: *p* < 0.001, PDQ-39: *p* < 0.001); overall cognition was not significantly different from preoperative (MMSE: *p* = 0.275, MoCA: *p* = 0.913), although language function was significantly impaired compared to preoperative (MMSE: *p* = 0.004, MoCA: *p* = 0.009); depression and anxiety symptoms also improved significantly (HAMD: *p* < 0.001, HAMA: *p* < 0.001) and the depression score correlated significantly with motor (preoperative: *r* = 0.493, *p* = 0.009), disease duration (preoperative: *r* = 0.519, *p* = 0.006; postoperative: *r* = 0.406, *p* = 0.036) and H&Y (preoperative: *r* = 0.430, *p* = 0.025; postoperative: *r* = 0.387, *p* = 0.046); total anxiety scores were also significantly correlated with motor (preoperative: *r* = 0.553, *p* = 0.003; postoperative: *r* = 0.444, *p* = 0.020), disease duration (preoperative: *r* = 0.417, *p* = 0.031), PDQ-39 (preoperative: *r* = 0.464, *p* = 0.015) and H&Y (preoperative: *r* = 0.440, *p* = 0.022; postoperative: *r* = 0.526, *p* = 0.005).

**Conclusion:**

STN-DBS is a safe and effective treatment for YOPD. The mood improved significantly, and overall cognition was not impaired, were only verbal fluency decreased but did not affect the improvement in quality of life.

## Introduction

Parkinson’s disease (PD) is a progressive neurodegenerative disease that affects the central nervous system. Its pathogenesis is associated with a variety of pathologies including neuroinflammation due to misfolding of α-synuclein, mitochondrial dysfunction and neurotransmitter-driven alterations in the neural network of the brain (Titova et al., 2017). It is characterized by typical motor symptoms such as tremors, rigidity, bradykinesia, postural gait disturbances, and a range of non-motor symptoms (NMS; Duncan et al., 2014). With the current aging of the population, it is estimated that by 2030, China will account for about 50% of the world’s PD patients (Dorsey et al., 2007). Age of onset is highly significant in various neurodegenerative diseases, as it correlates with the disease’s clinical phenotype and progression. Parkinson’s disease can be divided into two subtypes according to the time of disease onset: young-onset Parkinson’s disease (YOPD) and late-onset Parkinson’s disease (LOPD). The criteria for classifying patients with YOPD and LOPD are not uniform and are mainly based on age of onset. The maximum age reported for YOPD ranges from 40 to 55 years (Butterfield et al., 1993; Schrag et al., 2003; Mehanna and Jankovic, 2019; Do et al., 2023). Some scholars use 40 years of age as the dividing point, while others refer to patients with onset before 50 years of age as YOPD and those with onset after 50 years of age as LOPD (Mahale et al., 2014; Liu et al., 2015; Mehanna and Jankovic, 2019; Kaiyrzhanov et al., 2021). The mechanisms and manifestations of the two types of Parkinson’s disease are not the same. It was found that most patients with PINK1 gene mutations had an earlier age of onset, mostly between 32 and 48 years old (Huang et al., 2023). In addition, YOPD patients taking levodopa had a lower risk of dementia and gait disorders compared with LOPD patients, and there are also differences in the incidence and severity of various NMS (Mehanna and Jankovic, 2019).

As PD continues to be explored, levodopa and other dopaminergic drugs have been widely used. However, as the disease progresses in the mid to late stages, the effectiveness of drug therapy decreases, and long-term high-dose application eventually leads to motor complications and the more effective deep brain stimulation (DBS) has emerged due to this treatment bottleneck (Mohr et al., 2011; Warren Olanow et al., 2013; Asahi et al., 2014). The ventral intermediate nucleus (VIM), the globus pallidus internal (GPI), and the subthalamic nucleus (STN) are the most commonly used clinical targets. STN is the most chosen target in the current DBS treatment of PD because of its ability to control motor symptoms relatively comprehensively (Kleiner-Fisman et al., 2006). The research suggests that patients with STN-DBS have improved motor symptoms in the short and long term (Witt et al., 2008; Fasano et al., 2010; Wu et al., 2014). However, the physiological basis of DBS surgery for Parkinson’s disease is not well understood, the degree of improvement in DBS varies between patients, and minimal data are focusing on the role of age at the onset of PD on the outcome of patients treated for STN-DBS. More significant improvements in the motor have been reported in YOPD compared to LOPD with STN-DBS, which may be related to the fact that patients with YOPD have slower disease progression (Kempster et al., 2007; Otaka et al., 2010; Tsai et al., 2013). Based on these results, STN-DBS may be more effective if surgery is performed early in the onset of PD (Merola et al., 2012; Schuepbach et al., 2013).

Although DBS can dramatically improve motor symptoms, its effect on NMS in PD patients has long been overlooked. In addition to motor symptoms, PD patients also suffer from various NMS, such as hyposmia, depression/anxiety, cognitive dysfunction, sleep disturbances, and constipation. Braak proposed that NMS frequently occurs in all stages of PD and is a pre-motor symptom (Braak et al., 2003). NMS has become a severe condition that plagues patients after motor symptoms have been controlled.

Cognitive impairment and altered mood symptoms in NMS are more prevalent in PD, where patients develop cognitive decline in areas such as memory, executive ability, and language, as well as anxiety and depressive symptoms as the disease progresses. There has been increasing interest in the effects of DBS on cognition and mood in PD patients, but the findings remain controversial. Some scholars believe that PD patients have reduced memory and verbal fluency after DBS (Weaver et al., 2009); while another study suggests that patients’ memory improves after surgery, with only verbal fluency and executive function declining (Halpern et al., 2009); even as 32% of patients in the STN-DBS surgery group were observed to transform into dementia after 2 years of follow-up in one study (Williams et al., 2011). There are also conflicting studies on mood state, with some reporting that bilateral STN stimulation significantly improves anxiety and that this improvement is more often seen in patients with the more excellent recovery of motor function in response to stimulation (Fabbri et al., 2017). Nevertheless, randomized studies with unilateral STN or GPi-DBS have found that patients’ anxiety symptoms were worse at 2, 4, 6, and 12 months postoperatively than at baseline (Okun et al., 2014); other studies have shown no significant Impairment post-DBS (Wang et al., 2016; Sarno et al., 2019). Meanwhile, in various studies on the effect of depression in PD patients, the same differing results of improvement or no influence or even worsening were presented (Deuschl et al., 2006; Mehta and Sethi, 2009; Pariwatcharakul et al., 2013; Chandran et al., 2014).

In conclusion, the results of the studies on NMS, such as the cognition and mood of PD patients with STN-DBS, are still diverse, and most of the subjects in these reports are LOPD, while there are few kinds of research on YOPD. In contrast to LOPD patients with similar disease duration and severity, YOPD patients have greater social and family responsibilities or stresses and can be more concerned about their physical status. There is also growing evidence that increasing the age of onset of PD is associated with low cognition (Levy, 2007; Wickremaratchi et al., 2009). The relationship between the age of onset in depression and anxiety states has been inconsistently shown in various studies (Pagano et al., 2016; Hu et al., 2018; Park et al., 2018). Therefore, using YOPD patients as study subjects is even more crucial. To this end, we conducted this clinical study to observe the postoperative cognitive function, mood, and motor symptoms of YOPD patients who received bilateral STN-DBS to investigate their impact and help doctors make the best clinical decisions to optimize the neuromodulation treatment of PD.

## Materials and methods

### Participants

**Figure 1:**
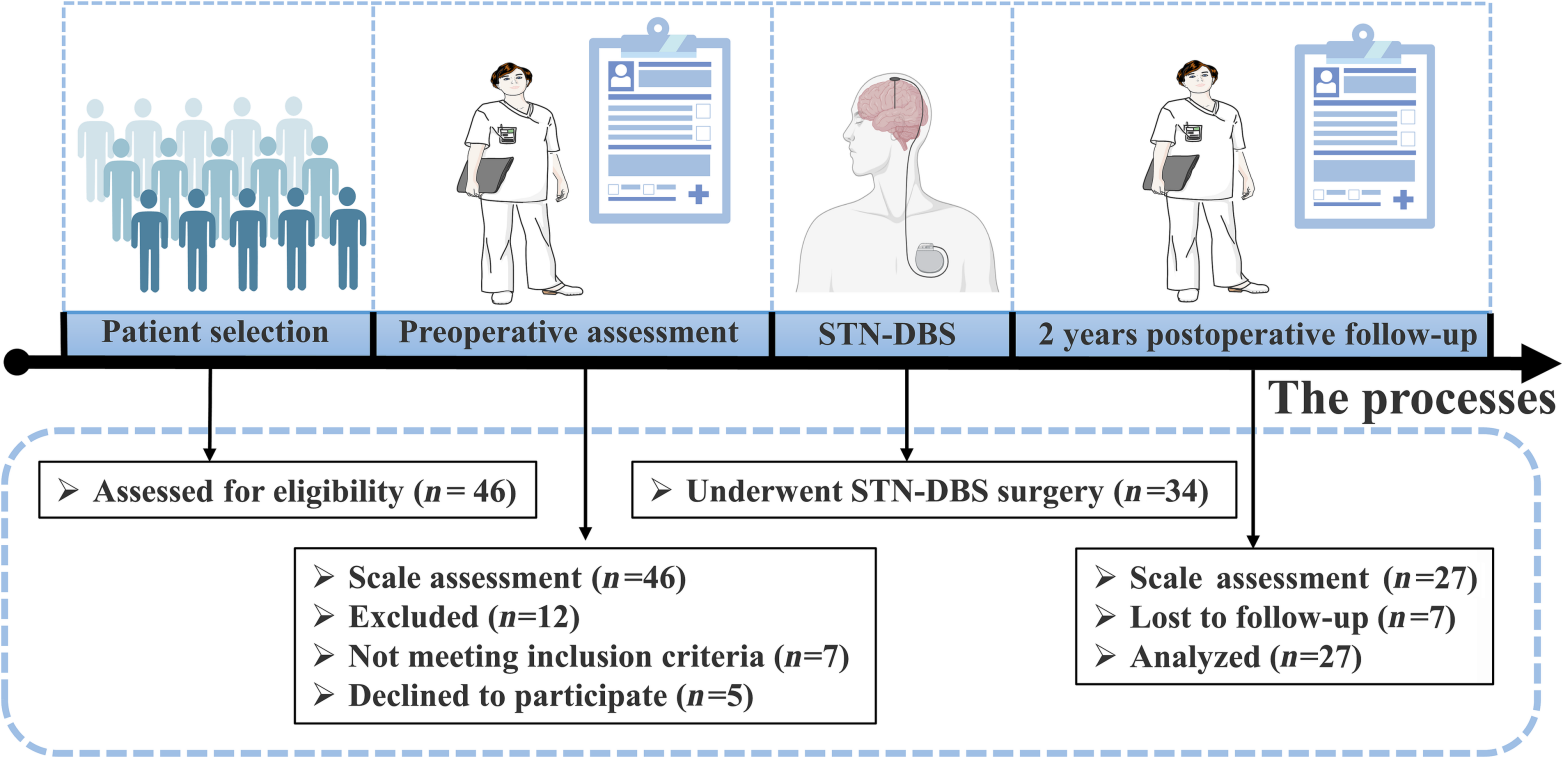
Schematic diagram of YOPD patient screening and study design. 27 of the 34 YOPD patients who underwent STN-DBS completed 2 years postoperative follow-up and were evaluated by professional clinical staff on the relevant scales.

This study included patients with YOPD who underwent bilateral STN-DBS at the General Hospital of the Chinese People’s Liberation Army from May 2019 to September 2021. The 46 patients with YOPD who entered the initial screening were evaluated with separate scale tests, and 12 were excluded because they did not meet the inclusion criteria or refused to participate in this study. The remaining 34 patients with YOPD underwent STN-DBS, of which 27 completed the 2-year follow-up and were included in the study (Figure 1).There were 9 female cases, overall age of onset of 39.48 ± 6.24 years, duration of disease of 8.96 ± 2.78 years, and 11.70 ± 3.94 years of education (Table 1). The inclusion criteria for having STN-DBS surgery were as follows: fulfilling the diagnostic criteria for Parkinson’s disease (Postuma et al., 2015); age < 50 years at onset of PD (YOPD); favorable response to levodopa on the Unified Parkinson’s Disease Rating motor assessment (UPDRS III; >30% improvement); no structural lesions on brain magnetic resonance imaging (MRI); and no contraindications to neurosurgery. The local ethics committee approved the study protocol, and eligible patients signed an informed consent form before entering the study.

**Table 1:** Clinical characteristics of YOPD patients (*n* = 27).

Demographic and clinical variable	Baseline, Mean ± SD
Sex (male/female)	18/9
Age (years)	48.44 ± 4.85
Age at PD onset (years)	39.48 ± 6.24
Duration of the disease (years)	8.96 ± 2.78
Education (years)	11.70 ± 3.94
The total score of UPDRS	
UPDRS I	12.33 ± 5.42
UPDRS II	15.85 ± 5.98
UPDRS III(Med-off)	52.67 ± 12.17
UPDRS III(Med-on)	22.89 ± 6.14
UPDRS VI	8.04 ± 4.00
LEDD (mg/day)	
Baseline	818.19 ± 240.51
Follow-up	369.48 ± 109.46

### Surgical procedure

**Figure 2:**
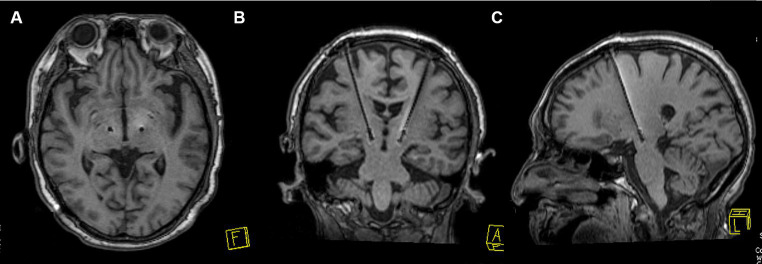
STN-DBS electrode position verification image. **(A–C)** An MRI example of electrode placement in the STN is shown to verify the accuracy of electrode placement.

YOPD Patients were placed in a stereotactic head frame (Leksell Model F head frame) under local anesthesia. Stereotactic magnetic resonance imaging (MRI) was performed with a 3 T scanner (Siemens Espree), scanning to obtain a plane containing the anterior commissure (AC) and posterior commissure (PC). Coronal and sagittal images were taken orthogonal to the axial image. The anatomical target coordinates of the STN were located 5 mm inferior to the midpoint of the AC-PC line, 2 mm posterior, and 12 mm laterally. Targets were adjusted to the center of the STN due to individual differences in STN morphology. The entry point was determined based on MRI images with target coordinates, and intraoperative microelectrode recording was used to place the DBS electrode (Medtronic 3,389 s, Medtronic, or PINS L301, PINS Medical Co.). Awareness of the patients and external stimulators (Programmer 8,840) for testing verbal feedback and physical activity. This was followed by intraoperative MRI (iMRI) to verify the accuracy of electrode placement. If the iMRI showed accurate electrode placement, the implantable pulse generator (IPG; Medtronic Activa RC, Medtronic, or PINS G102RZ, PINS Medical Co.) was placed on the chest; else, the coordinates were adjusted to ensure electrode position. Postoperatively, the MRI and CT scans were performed to revalidate the accuracy of the targets and to exclude the risk of intracranial hematoma (Figure 2). Postoperative stimulation was performed 1–2 weeks after surgery to select the optimal stimulation contacts and parameters to achieve satisfactory patient improvement.

### Assessment methods

The Patients with STN-DBS were assessed for H&Y, quality of life, motor symptoms, cognition, depression, anxiety, daily levodopa equivalent dose (LEDD), and other relevant clinical symptoms 1 week before and 2 years after surgery (Tomlinson et al., 2010). All motor symptoms were evaluated in the medication-on (Med-on) and medication-off (Med-off) status; the preoperative Med-on was the period of optimal symptom control after taking PD medication, and the preoperative Med-off was when PD medication had been stopped for at least 12 h. The postoperative Med-off means that the pulser was working optimally and the medication had been stopped for 12 h or more, while the postoperative Med-on refers to the best working condition of the pulser and the peak performance after medication. A clinical professional assessed the cognitive and mood symptoms during the “on” state of medication.

Motor function: The Unified Parkinson’s Disease Rating Scale (UPDRS) evaluated patients’ mental, behavioral, emotional, activities of daily living, motor, and complications, and consists of four subscales: UPDRS I, UPDRS II, UPDRS III, and UPDRS VI. Each item was scored on a five-point scale of 0, 1, 2, 3, and 4, with the higher the score, the more severe the PD symptoms (Martínez-Martín et al., 1994). The UPDRS III primarily evaluated the motor abilities of YOPD patients, and the UPDRS III subscales consisted of bradykinesia, tremor, rigidity, and axial symptoms. The axial subscore was calculated as the sum of speech, gait, postural stability, neck rigidity, posture, and arising from a chair, with the first three items focused mainly on in the study. The H&Y was used to assess the severity of the disease and the scores were also inversely related to the function.

Quality of life: The 39-Item Parkinson’s Disease Questionnaire (PDQ-39) was used to assess patients’ quality of life in terms of their ability to perform activities of daily living and motor function. Thirty-nine questions were asked on eight dimensions, including mobility, activities of daily living (ADL), emotional well-being, stigma, social support, cognition, communication, and bodily discomfort (Hamilton, 1960; Peto et al., 1995). The scores were inversely related to the quality of life.

Cognition: The patient’s cognitive function was initially assessed using the Mini-mental state examination (MMSE), which has five components: directionality, memory, attention and calculation, delayed recall, and language, for a total of 30 points, with the advantage of high specificity (Folstein et al., 1975); the cognitive state of the YOPD patient was further evaluated using the Montreal Cognitive Assessment (MoCA). The MoCA has seven components: Visual space and execution, picture recognition, attention and calculation, language, abstraction, delayed recall, and directionality, for a total of 30 points, and has the strength of sensitivity (Nasreddine et al., 2005). The scores were proportional to symptoms.

Mood state: Hamilton depression scale (HAMD) and Hamilton anxiety scale (HAMA) assess the depression and anxiety states of YOPD patients (Hamilton, 1959). HAMD consists of 24 questions with seven dimensions, and the criteria for determining the results are: <8 scores: no depression; 8–20 scores: possible depression; >20 scores: mild to moderate depression; >35 scores: severe depression. HAMA is mainly divided into two domains of somatic anxiety and mental anxiety, with 14 items, and the criteria are: <7 scores: no anxiety; >7 scores: possible anxiety; >14 scores: definitely anxiety; >21 scores: apparent anxiety; >29 scores: severe anxiety. The scores were inversely related to the quality of life.

### Statistical analyses

Statistical analysis was performed using SPSS13.0 and origin2021 for statistical processing and descriptive analysis. Changes in each follow-up indicator before and after STN-DBS surgery were expressed in the form of improvement rate as a percentage, improvement rate (%) = absolute value of (postoperative score − preoperative score) × 100% / preoperative score. Measures that conformed to a normal distribution were analyzed using the paired t-test. In contrast, measures that did not conform to a normal distribution were analyzed using the Wilcoxon non-parametric rank sum test. Correlations were analyzed using Pearson correlation, with *p* < 0.05 indicating a statistically significant difference.

## Results

A total of 27 patients with PD were included in the study, of whom 18 were male, overall mean age of 48.44 ± 4.85 years. As shown in Table 1, the mean preoperative duration of the disease was 8.96 ± 2.78 years, the mean age of onset was 39.48 ± 6.24, and the UPDRS I, UPDRS II, and UPDRS IV scores were 12.33 ± 5.42, 15.85 ± 5.98, and 8.04 ± 4.00. The motor subscale UPDRS III Med-on and off scores were 52.67 ± 12.17 and 22.89 ± 6.14. all patients underwent bilateral STN-DBS surgery, received preoperative levodopa medication, and had >30% effectiveness. Two years after surgery, LEDD decreased from 818.19 ± 240.51 mg preoperatively to 369.48 ± 109.46 mg, a 54.84% reduction (*p* < 0.001).

All data are expressed as means ± standard deviations; YOPD, young onset Parkinson’s disease; UPDRS I, Unified Parkinson’s Disease Rating Scale part I (non-motor) score; UPDRS II, Unified Parkinson’s Disease Rating Scale part II (activities of daily living) score; UPDRS III, Unified Parkinson’s Disease Rating Scale part III (motor) score; UPDRS VI, Unified Parkinson’s Disease Rating Scale part VI (complications) score; Med-on, evaluation performed under the pharmacological effect of dopaminergic therapies; Med-off, evaluation performed at least 12 h after the last levodopa dose; LEDD, levodopa equivalent daily dose.

### Motor outcome

The 27 YOPD patients with STN-DBS in this study showed significant improvement in H&Y and motor function compared to the corresponding period before surgery, both in the Med-on and Med-off state (*p* < 0.001). Table 2 shows, in the Med-off state, the total UPDRS III scores were 52.67 ± 12.17 after surgery with only STN-DBS treatment, which was reduced significantly compared to the total preoperative scores of 25.11 ± 3.95 (*p* < 0.001), with an improvement rate of 52.32%. Among the subscales, the improvement rates of bradykinesia, tremor, and rigidity were 58.75% (*p* < 0.001), 58.52% (*p* < 0.001), and 44.79% (*p* < 0.001), respectively. The improvement rate of axial was 37.16% (*p* < 0.001), but speech function was significantly impaired (*p* = 0.034). In the Med-on state, the total UPDRS III scores at follow-up were 22.89 ± 6.14 compared to the total preoperative scores of 16.19 ± 3.25 (*p* < 0.001), with the combination of STN-DBS treatment and medication. Rigidity, tremor and bradykinesia all improved more (*p* < 0.001). However, no significantly improved axial symptoms were observed compared to the preoperative Med-on period (*p* = 0.061). In addition, H&Y was greatly reduced in the postoperative Med-off and Med-on states compared to the corresponding preoperative period (2.26 ± 0.32 vs. 3.24 ± 0.76, and 1.83 ± 0.24 vs. 2.28 ± 0.42, *p* < 0.001). The total score revealed that YOPD patients had the best results in the Med-on state 2 years after surgery, suggesting that the combination of DBS and medication is more productive than DBS alone. That postoperative anti-Parkinsonian medication is still needed to achieve the greatest results.

**Table 2:** YOPD patients’ motor scores in Med-off and Med-on conditions before and after bilateral STN stimulation (*n* = 27).

Motor symptoms	Range	Med-off		Med-on		*p* value	
		Baseline	Follow-up	Baseline	Follow-up	Med-off	Med-on
UPDRS III							
Total	0–132	52.67 ± 12.17	25.11 ± 3.95	22.89 ± 6.14	16.19 ± 3.25	<0.001^***^	<0.001^***^
Tremor	0–40	9.74 ± 4.47	4.04 ± 1.51	3 (2, 4)	2 (1, 3)	<0.001^***^	0.001^**^
Rigidity	0–20	10.07 ± 2.50	5.56 ± 1.76	6.30 ± 3.23	3.44 ± 1.28	<0.001^***^	<0.001^***^
Bradykinesia	0–48	23.78 ± 7.67	9.81 ± 1.84	8.93 ± 2.13	6.96 ± 1.56	<0.001^***^	<0.001^***^
Total axial	0–24	9.07 ± 3.27	5.70 ± 1.86	4.52 ± 2.21	3.93 ± 1.41	<0.001^***^	0.061
Speech	0–4	1 (1, 1)	1 (1, 2)	1 (1, 1)	1 (1, 1)	0.034^*^	0.180
Postural stability	0–4	1 (1, 2)	1 (0, 2)	0 (0, 1)	0 (0, 1)	<0.001^***^	0.046^*^
Gait	0–8	2(2, 2)	1 (1, 2)	1 (0, 2)	1 (0, 1)	<0.001^***^	0.033^*^
**H&Y**	0–5	3.24 ± 0.76	2.26 ± 0.32	2.28 ± 0.42	1.83 ± 0.24	<0.001^***^	<0.001^***^

^*^p < 0.05, ^**^p < 0.01, ^***^p < 0.001.

Normal data are expressed as means ± standard deviations; Nonnormal data are expressed as median (lower quartile, upper quartile); YOPD, young onset Parkinson’s disease; UPDRS III, Unified Parkinson’s Disease Rating Scale part III (motor) score; H&Y, Hoehn and Yahr stage; Med-on, evaluation performed under the pharmacological effect of dopaminergic therapies; Med-off, evaluation performed at least 12 h after the last levodopa dose; A probability value of *p* < 0.05 was considered significant. ^*^p < 0.05, ^**^p < 0.01, ^***^p < 0.001.

### Quality of life

The quality of daily life for patients with YOPD was evaluated by the PDQ-39 (Table 3). This study revealed that the total PDQ-39 scores at baseline and 2 years postoperatively were 45.07 ± 14.41 and 27.52 ± 5.86, representing an improvement of 38.94% (*t* = 9.128, *p* < 0.001). The enhancement in the motor ability and ADL components of the PDQ-39 scores directly resulted from the patients’ improved motor symptoms postoperatively. The PDQ-39 subscales demonstrated the greatest increase in motor activity (*z* = −4.482, *p* < 0.001), and in the other subscales, ADL (*z* = −4.380, *p* < 0.001), emotional well-being (*z* = −4.566, *p* < 0.001), stigma (*t* = 3.770, *p* = 0.001) and bodily discomfort (*z* = −3.501, *p* < 0.001) scores also all showed significant improvements compared to the preoperative period. There was, however, a downward trend in social support (*z* = −1.732, *p* = 0.083), cognition (*t* = 1.894, *p* = 0.069), and communication (*t* = 0.811, *p* = 0.425) at the 2-year postoperative follow-up, but it was not statistically significant.

**Table 3:** Quality of life, cognitive and mood scores of YOPD patients before and after bilateral STN stimulation (*n* = 27).

	Range	Baseline	Follow-up	t/z value	*p* value
PDQ-39					
Total score	0–156	45.07 ± 14.41	27.52 ± 5.86	9.128	<0.001^***^
Mobility	0–40	9 (5, 15)	4 (3, 6)	−4.482	<0.001^***^
Activities of daily living	0–24	8 (5, 9)	4 (3, 6)	−4.380	<0.001^***^
Emotional well-being	0–24	6 (5, 9)	3 (2, 5)	−4.566	<0.001^***^
Stigma	0–16	6.67 ± 2.40	4.81 ± 1.59	3.770	0.001^**^
Social support	0–12	1 (1, 2)	1 (1, 2)	−1.732	0.083
Cognition	0–16	3.96 ± 1.26	3.52 ± 0.98	1.894	0.069
Communication	0–12	2.78 ± 1.05	2.63 ± 0.88	0.811	0.425
Bodily discomfort	0–12	3(2, 5)	2 (2, 3)	−3.501	<0.001^***^
MMSE					
Total score	0–30	28.11 ± 1.78	27.81 ± 1.55	1.114	0.275
Directionality	0–10	10.00 ± 0.00	9.93 ± 0.27	1.442	0.161
Memory	0–3	2.85 ± 0.36	2.93 ± 0.27	−1.442	0.161
Attention and calculation	0–5	4.19 ± 1.00	4.30 ± 0.82	−0.769	0.449
Delayed recall	0–3	2.56 ± 0.64	2.63 ± 0.56	−0.527	0.602
Language	0–9	8.52 ± 0.64	8.04 ± 0.71	3.118	0.004^**^
MOCA					
Total score	0–30	25.59 ± 2.66	25.55 ± 1.97	0.110	0.913
Visual space and execution	0–5	4.19 ± 0.79	4.26 ± 0.71	−1.000	0.327
Picture recognition	0–3	2.78 ± 0.51	2.70 ± 0.61	1.442	0.161
Attention	0–6	5.37 ± 0.79	5.33 ± 0.73	0.254	0.802
Language	0–3	2.19 ± 0.56	1.89 ± 0.58	2.842	0.009^**^
Abstraction	0–2	1.85 ± 0.36	1.81 ± 0.40	0.570	0.574
Delayed recall	0–5	3.37 ± 0.84	3.63 ± 0.84	−1.763	0.090
Directionality	0–6	5.85 ± 0.36	5.93 ± 0.27	−0.811	0.425
HAMD					
Total score	0–96	15.07 ± 3.57	9.41 ± 1.31	8.909	<0.001^***^
Anxiety/somatization	0–24	3 (3, 5)	2 (2, 4)	−2.535	0.011^*^
Weight	0–4	0 (0, 1)	0 (0, 0)	−1.069	0.285
Cognitive disturbance	0–24	1 (0, 2)	0 (0, 1)	−3.562	<0.001^***^
Diurnal variation	0–4	1 (1, 1)	0 (0, 1)	−2.714	0.007^**^
Retardation	0–16	3 (2, 4)	2 (1, 2)	−4.409	<0.001^***^
Sleep disturbance	0–12	3 (2, 4)	2 (2, 3)	−3.158	0.002^**^
Hopelessness	0–12	3 (2, 4)	2 (1, 3)	−3.934	<0.001^***^
HAMA					
Total score	0–56	10.89 ± 2.49	6.74 ± 2.23	6.056	<0.001^***^
Somatic anxiety	0–28	4.19 ± 1.27	2.59 ± 0.84	8.522	<0.001^***^
Psychic anxiety	0–28	7 (4, 9)	4 (3, 5)	−3.926	0.001^**^

Normal data are expressed as means ± standard deviations; Nonnormal data are expressed as median (lower quartile, upper quartile); YOPD, young onset Parkinson’s disease; PDQ-39, 39-Item Parkinson’s Disease Questionnaire; MMSE, Mini-mental state examination; MoCA, Montreal Cognitive Assessment; HAMD, Hamilton depression scale; HAMA, Hamilton anxiety scale; A probability value of *p* < 0.05 was considered significant. ^*^p < 0.05, ^**^p < 0.01, ^***^p < 0.001.

### Cognitive function

Using the MoCA and MMSE scales to assess the cognitive state of YOPD patients before and after surgery (Table 3), the total MMSE scores were 28.11 ± 1.78 and 27.81 ± 1.55 at baseline and follow-up (*t* = 1.114, *p* = 0.275). In the MMSE scores for each cognitive dimension, YOPD patients’ directionality (*t* = 1.442, *p* = 0.161), memory (*t* = −1.442, *p* = 0.161), attention and calculation (*t* = −0.769, *p* = 0.449) and delayed recall (*t* = −0.527, *p* = 0.602) were not compared to preoperative statistically significant, with only language showed a significant decrease (*t* = 3.118, *p* = 0.004). 12 of the 27 patients in this group (44%) received an impact on language, mainly in language fluency. Again a significant decline in all cognitive domains of MoCa was found only in language function in comparison to preoperative (*t* = 2.842, *p* = 0.009), with 9 patients (33%) having lower scores postoperatively. Outcomes for overall cognition were consistent with the MMSE, with total MoCA scores of 25.59 ± 2.66 and 25.55 ± 1.97 pre- and postoperatively, with no significant differences found (*t* = 0.110, *p* = 0.913). Also Visual space and execution ability (*t* = −1.000, *p* = 0.327), Picture recognition (*t* = 1.442, *p* = 0.161), attention (*t* = 0.254, *p* = 0.802), abstraction (*t* = 0.570, *p* = 0.574), delayed recall (*t* = −1.763, *p* = 0.090), Directionality (*t* = −0.811, *p* = 0.425) scores also all did not change meaningfully.

### Mood state

As shown in Table 3, the outcomes of the HAMD scores for most dimensions before and after surgery were dramatically reduced for anxiety/somatization (*z* = −2.535, *p* = 0.011), cognitive disturbance (*z* = −3.562, *p* < 0.001), diurnal variation (*z* = −2.714, *p* = 0.007), retardation (*z* = −4.409, *p* < 0.001), Sleep disturbance (*z* = −3.158, *p* = 0.002), and Hopelessness (*z* = −3.934, *p < 0.001*). The total HAMD score was 15.07 ± 3.57 vs. 9.41 ± 1.31 (*t* = 8.909, *p* < 0.001) and an improved rate of 37.56% overall. 3 of the 27 surgical patients had a mild to moderate depressive state (score > 20), and the remainder were possibly depressed (score: 8–20). Postoperatively, most patients showed a significant reduction in symptoms. The HAMA results reveal an effective postoperative anxiety improvement of 38.11% in total (*t* = 6.056, *p* < 0.001), from 10.89 ± 2.49 preoperatively to 6.74 ± 2.23. Both subscales of somatic anxiety (*t* = 8.522, *p* < 0.001) and Psychic anxiety (*z* = −3.926, *p* = 0.001) were also significantly decreased. At baseline, 2 of 27 patients was determined to have anxiety symptoms (score > 14), and 25 were possible anxiety (score > 7). The number of patients with postoperative anxiety was reduced to 13, and the other 14 had no anxiety symptoms (score < 7). Compared with the baseline, the HAMA and HAMD scores of the YOPD patients in this group decreased significantly during the follow-up, and the mood disorders were relieved.

### Overall outcome and correlation analysis

**Figure 3:**
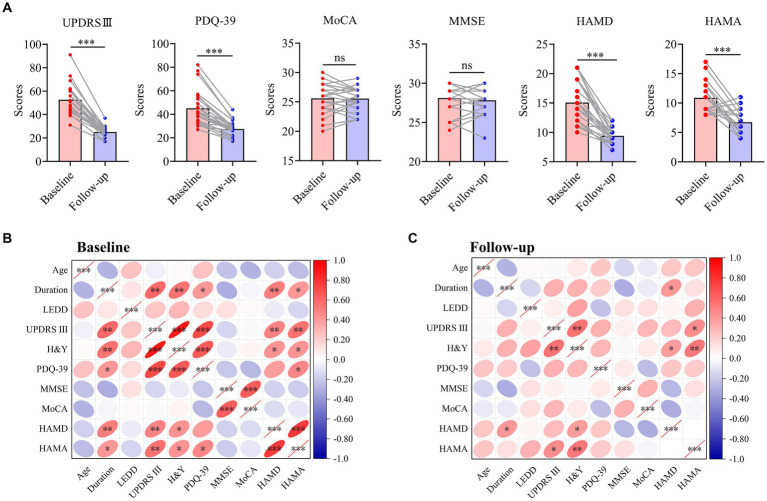
Overall outcome and correlation analysis of patients with YOPD. **(A)** The change of clinical outcomes in patients with YOPD at baseline and 2-year follow-up. The pink indicates each clinical symptom score for YOPD patients at baseline; The blue indicates each clinical symptom score for YOPD patients at 2-year follow-up. **(B,C)** Heat map of the correlation matrix between clinical variables at baseline and 2-year follow-up. YOPD, young onset Parkinson’s disease; UPDRS III, Unified Parkinson’s Disease Rating Scale part III (motor) score; PDQ-39, 39-Item Parkinson’s Disease Questionnaire; H&Y, Hoehn and Yahr stage; LEDD, levodopa equivalent daily dose; MMSE, Mini-mental state examination; MoCA, Montreal Cognitive Assessment; HAMD, Hamilton depression scale; HAMA, Hamilton anxiety scale; A probability value of *p* < 0.05 was considered significant. ^*^p < 0.05, ^**^p < 0.01, ^***^p < 0.001, ns: not significant.

YOPD patients showed significant increases in motor or mood (*p* < 0.001) and further improvements in quality of life after STN-DBS, except for no significant changes in overall cognition, which confirmed the effectiveness and safety of STN-DBS for YOPD patients (Figure 3A). Pearson correlation analysis further explored the correlation between cognitive ability or mood and various other variables at different time points (Figures 3B,C; Supplementary Table 1). Preoperative depressive symptoms were significantly correlated with the disease duration (*r* = 0.519, *p* = 0.006), motor ability (*r* = 0.493, *p* = 0.009) and H&Y (*r* = 0.430, *p* = 0.025), and postoperatively also correlated with disease duration (*r* = 0.406, *p* = 0.036) and H&Y (*r* = 0.387, *p* = 0.046). Preoperative anxiety symptoms were significantly correlated with the disease duration (*r* = 0.417, *p* = 0.031), motor ability (*r* = 0.553, *p* = 0.003), H&Y (*r* = 0.440, *p* = 0.022) and PDQ-39 (*r* = 0.464, *p* = 0.015), and improvement in postoperative anxiety was significantly correlated with improved motility (*r* = 0.444, *p* = 0.020) and H&Y (*r* = 0.526, *p* = 0.005). Nevertheless, no correlation was seen between postoperative total cognition and other variables (*p* > 0.05).

## Discussion

DBS is rapidly evolving; among them, bilateral STN-DBS has become the primary choice for PD. However, several studies have not reached consistent conclusions regarding the postoperative effects of NMS. Many patients, especially YOPD, are concerned about the adverse impact on other NMS while improving motor symptoms. Thus, this study investigated the effects of DBS on NMS with a high prevalence of YOPD patients, such as cognition, anxiety, and depression.

This study is consistent with previous studies regarding improving motor and quality of life (Fasano et al., 2010; Lahtinen et al., 2020; Bove et al., 2021; Bezdicek et al., 2022; Golfrè Andreasi et al., 2022; Zeng et al., 2023). It is shown that postoperative Med-off treatment with only STN-DBS improved PD motor compared with preoperative Med-off status. All motor scores improved 2 years after surgery (tremor 58.52%, rigidity 44.79%, bradykinesia 58.75%, and axial symptoms 37.16%), demonstrating that DBS alone can significantly enhance various motor symptoms in patients with YOPD, and the effect is sustained. However, STN-DBS has a limited effect on the axial of YOPD patients. A further 5-year long-term follow-up study showed worse scores for axial than preoperatively, which may also be related to progressive disease progression (Fasano et al., 2010). The rate of improved postoperative Med-on status in YOPD patients was significantly higher than at baseline Med-off, suggesting that the combination of DBS and drugs maximized efficacy and significantly reduced postoperative medication doses. Significant progress in overall PDQ-39 scores, consistently with other studies (Lee et al., 2006; Büttner et al., 2019; Schuepbach et al., 2019; Chen et al., 2023). Changes in PDQ-39 were significantly associated with improvements in motor function, further substantiating the effectiveness of STN-DBS and laying the foundation for slowing NMS in YOPD patients.

Regarding cognition, some studies have argued that long-term DBS treatment may cause abnormal cognitive function (Aybek et al., 2007; Williams et al., 2011; David et al., 2020; Pal et al., 2023). Nevertheless, studies with up to 2 years of follow-up did not find any cognitive changes in patients (Georgiev et al., 2021). Several studies have shown that cognition, especially verbal fluency, is degraded in PD patients after DBS, which may be related to the different criteria for the inclusion of patients in each center, the different cognitive rating scales used, and the progression of PD itself (Hogg et al., 2017; Gratwicke et al., 2018; Kawaguchi et al., 2020). This study indicates that 2 years after STN-DBS in YOPD patients, both MoCA and MMSE total scores were not statistically significant compared to preoperative, there were no significant changes in each cognitive subscale, and only language function was impaired, again mainly in the form of a significant lowering of verbal fluency, consistent with the relevant papers (Duncan et al., 2014b; Demeter et al., 2017; Tröster et al., 2017; Hyder et al., 2021; John et al., 2021). However, no improvements in Visual space function and attention reported in other studies were found. In contrast, the present study found no significant reduction in total cognitive performance in patients before and after DBS. The reasons could be: (1) this study is a two-year follow-up study and lacks longer-term follow-up data; (2) Differences in the type of anesthesia taken during surgery in different patients between the studies may have an impact on the postoperative outcome, leading to inconsistent results (Brodsky et al., 2017; Blasberg et al., 2018; Jiang et al., 2021). (3) Patients with YOPD are younger, and it has been reported that intracranial tau protein levels are lower in YOPD patients than in LOPD. The increase in tau protein is associated with disrupted neural network connections in the brain and progressive degeneration of the substantial nigra (Gomperts et al., 2016). (4) And there exists a significantly lower level of Aβ42 in the cerebrospinal fluid of PD patients compared with the average population, which leads to the aggravation of intracranial amyloid plaque deposition and becomes a strong predictor for the assessment of cognitive impairment in PD patients (Alwardat et al., 2019). It can be speculated that the level of Aβ42 is further decreased in LOPD patients compared with YOPD patients. This may be one of the reasons for the absence of significant overall cognitive impairment in YOPD after surgery in this study. (5) The intervention of DBS may affect the corticobasal ganglia loop and alter the output from the basal ganglia to the frontal lobe. This mechanism is mainly associated with impaired verbal fluency(Manes et al., 2014). Our study showed that some patients had language fluency Impact after surgery. However, it did not affect the total cognitive level, so STN-DBS is generally safe for YOPD, and that mild language fluency decline is not a contraindication to STN-DBS surgery.

Depression and anxiety are common in patients with PD, and the factors associated with their occurrence are equally controversial. Several studies have identified neurotransmitter abnormalities associated with developing anxiety and depression in PD, such as decreased dopamine levels and abnormal secretion of neurotransmitters, including adrenaline and 5-hydroxytryptamine (Remy et al., 2005; Prediger et al., 2012). Neurofunctional imaging studies also suggest that PD and anxiety and depression may share the same impaired chemical pathways, so the role of DBS in the brain’s neural network cannot be ignored (Black et al., 2005; Wen et al., 2016; Carey et al., 2021). The effect of STN-DBS on mood in our study may also be related to the neural network system. However, the results of the studies on the impact of STN-DBS on the mood of PD patients are diverse, with some studies reporting improvement in motor function as well as anxiety and depression in PD patients after surgery (Lieberman, 2006; Couto et al., 2014; Fabbri et al., 2017; Chuquilín-Arista et al., 2020; Santos-García et al., 2020; Cartmill et al., 2021). Another part of the study showed no change or a significant decrease in the mood at different times after surgery (Soulas et al., 2008; Chang et al., 2012; Seritan et al., 2021). The results of this study showed that 2 years after STN-DBS, the improvement rates of anxiety and depression in YOPD patients were 38.11 and 37.56%. The dimensions of anxiety/somatization, cognitive disturbance, diurnal variation, retardation, sleep disturbance, and hopelessness in HAMD were statistically distinct, and postoperative HAMA indicated that both somatic and psychic anxiety significantly progressed more than those before surgery. Analysis of the reasons: (1) It could be related to bilateral STN-DBS stimulation that alters brain structure and affects patients’ moods. The electrode contacts of STN-DBS can directly inhibit the limbic subregion of STN and indirectly affect the corticobasal ganglia limbic loop, and pulse stimulation of this loop can mediate mood responses and thus control the mood behavior of PD patients (Soulas et al., 2008). (2) STN-DBS may also have the effect of reducing mood disorder by improving the metabolism of neuronal cells and regulating the relevant transmitters that trigger depression and anxiety or regulating mood in PD patients by affecting other monoaminergic neural pathways, such as the serotonin-containing nucleus of the middle suture and the norepinephrine-containing nucleus of the blue spot (Gallagher and Schrag, 2012; Etiévant et al., 2015). (3) The correlation analysis implies that there was a correlation between the relief of depression and anxiety with the improved ability in patients’ daily life and motor, indicating that the enhancement of motor function and quality of life had an impact on the mood of YOPD. STN-DBS influences movement disorders by inhibiting the frontal lobe and further strengthens mood and neurological function (Castrioto et al., 2014; Combs et al., 2015).

## Limitations

There are some limitations in this study: (1) the follow-up period is short, only observed the changes of cognitive function, depression and anxiety after 2 years of bilateral STN-DBS treatment, and the patient population is YOPD, younger age, the potential impact produced may be reduced due to the younger age. Therefore, follow-up should be continued to observe the long-term effects of bilateral STN-DBS treatment on each clinical symptom in patients with YOPD. (2) The sample size of this study was limited, which may limit the accuracy of the statistical analysis. Further expansion of the sample size is needed to reduce the bias caused by insufficient sample size. (3) Scale tests are highly subjective, and more detailed cognitive neuropsychological tests should be used in addition to the MMSE and MoCA, and it is also recommended to incorporate objective assessment criteria, such as MR imaging, to help assess changes in brain structure and function before and after STN-DBS surgery. (4) More randomized controlled studies and further research to confirm the association between stimulation sites and neuropsychiatric disorders are needed in the future to help clinicians choose the best stimulation targets to guide better clinical decisions.

## Conclusion

In conclusion, STN-DBS for YOPD is a safe, minimally invasive, and effective treatment. This study revealed that YOPD patients had significantly lower postoperative levels of depression and anxiety, and that this improvement was in part associated with better motor and quality of life after STN-DBS. Regarding cognition, our results showed that STN-DBS causes cognitive decline in verbal fluency for YOPD patients. However, the MMSE and MoCA total scores indicated no significant impairment in overall cognitive level and did not affect improvement in quality of life. STN-DBS is a promising treatment modality for YOPD, and studying the effects of STN-DBS on the cognition and mood of YOPD will require larger sample sizes and longer-term randomized controlled trials at a later stage to select more accurate stimulation targets for DBS.

## Data availability statement

The original contributions presented in the study are included in the article/Supplementary material, further inquiries can be directed to the corresponding authors.

## Ethics statement

The studies involving humans were approved by Ethics Committee of the General Hospital of the Chinese People’s Liberation Army. The studies were conducted in accordance with the local legislation and institutional requirements. Written informed consent for participation in this study was provided by the participants’ legal guardians/next of kin. Written informed consent was obtained from the individual(s), and minor(s)’ legal guardian/next of kin, for the publication of any potentially identifiable images or data included in this article.

## Author contributions

JH and ZM contributed to conceptualization. JH and DL contributed to data collection and verification. HX contributed to the methodology. JH, HX, YC, TW, and ZM contributed to the data analysis. JH contributed to writing the original draft. ZM and KX contributed to writing, reviewing, and editing. All authors contributed to the article and approved the submitted version.
