# Supplementary material for: Effects of STN-DBS on cognition and mood in young-onset Parkinson’s disease: a two-year follow-up
Source: Front Aging Neurosci. 2024 Jan 16;15:1177889. doi: 10.3389/fnagi.2023.1177889 (PMC10824910; doi:10.3389/fnagi.2023.1177889)
Supplement: Supplementary file 1 [file Table_1.DOCX]

**Supplementary Table 1. Correlation of cognitive and mood with UPDRS Ⅲ, PDQ-39, and other clinical characteristics at different periods**

| **Factors** |  | **MMSE** | |  | **MoCA** | |  | **HAMD** | |  | **HAMA** | |
| --- | --- | --- | --- | --- | --- | --- | --- | --- | --- | --- | --- | --- |
|  |  | **Baseline** | **Follow-up** |  | **Baseline** | **Follow-up** |  | **Baseline** | **Follow-up** |  | **Baseline** | **Follow-up** |
| **Age** | ***r*** | −0.233 | −0.148 |  | −0.292 | −0.063 |  | −0.180 | 0.231 |  | −0.245 | 0.228 |
|  | ***p*** | 0.242 | 0.462 |  | 0.139 | 0.754 |  | 0.370 | 0.246 |  | 0.219 | 0.252 |
| **Duration** | ***r*** | −0.302 | −0.315 |  | −0.033 | −0.068 |  | **0.519** | **0.406** |  | **0.417** | 0.135 |
|  | ***p*** | 0.126 | 0.109 |  | 0.869 | 0.742 |  | **0.006**^**^ | **0.036**^*^ |  | **0.031^*^** | 0.502 |
| **LEDD** | ***r*** | 0.131 | 0.127 |  | 0.033 | 0.134 |  | −0.078 | −0.046 |  | −0.155 | 0.307 |
|  | ***p*** | 0.514 | 0.529 |  | 0.870 | 0.504 |  | 0.698 | 0.818 |  | 0.440 | 0.120 |
| **UPDRS Ⅲ** | ***r*** | −0.154 | 0.079 |  | −0.003 | 0.288 |  | **0.493** | 0.273 |  | **0.553** | **0.444** |
|  | ***p*** | 0.442 | 0.695 |  | 0.988 | 0.145 |  | **0.009^**^** | 0.168 |  | **0.003**^**^ | **0.020**^*^ |
| **H&Y** | ***r*** | −0.063 | −0.007 |  | 0.050 | 0.095 |  | **0.430** | **0.387** |  | **0.440** | **0.526** |
|  | ***p*** | 0.756 | 0.971 |  | 0.804 | 0.636 |  | **0.025^*^** | **0.046^*^** |  | **0.022^*^** | **0.005**^**^ |
| **PDQ−39** | ***r*** | −0.211 | 0.083 |  | −0.268 | −0.256 |  | 0.380 | 0.227 |  | **0.464** | 0.208 |
|  | ***p*** | 0.290 | 0.680 |  | 0.177 | 0.197 |  | 0.050 | 0.254 |  | **0.015^*^** | 0.298 |

YOPD, young onset Parkinson’s disease; UPDRS III, Unified Parkinson’s Disease Rating Scale part III (motor) score; PDQ-39, 39-Item Parkinson’s Disease Questionnaire; H&Y, Hoehn and Yahr stage; LEDD, levodopa equivalent daily dose; MMSE, Mini-mental state examination; MoCA, Montreal Cognitive Assessment; HAMD, Hamilton depression scale; HAMA, Hamilton anxiety scale; A probability value of *p* < 0.05 was considered significant. ^*^*p* < 0.05, ^**^*p* < 0.01, ^***^*p* < 0.001, ns: not significant.
